# Pulmonary disease detection and classification in patient respiratory audio files using long short-term memory neural networks

**DOI:** 10.3389/fmed.2023.1269784

**Published:** 2023-11-03

**Authors:** Pinzhi Zhang, Alagappan Swaminathan, Ahmed Abrar Uddin

**Affiliations:** ^1^College of Industrial and Systems Engineering, Georgia Institute of Technology, Atlanta, GA, United States; ^2^College of Computing, Georgia Institute of Technology, Atlanta, GA, United States

**Keywords:** artificial intelligence, neural networks, audio parsing, machine learning, pulmonary diagnostics, predictive analytics, lung disease

## Abstract

**Introduction:**

In order to improve the diagnostic accuracy of respiratory illnesses, our research introduces a novel methodology to precisely diagnose a subset of lung diseases using patient respiratory audio recordings. These lung diseases include Chronic Obstructive Pulmonary Disease (COPD), Upper Respiratory Tract Infections (URTI), Bronchiectasis, Pneumonia, and Bronchiolitis.

**Methods:**

Our proposed methodology trains four deep learning algorithms on an input dataset consisting of 920 patient respiratory audio files. These audio files were recorded using digital stethoscopes and comprise the Respiratory Sound Database. The four deployed models are Convolutional Neural Networks (CNN), Long Short-Term Memory (LSTM), CNN ensembled with unidirectional LSTM (CNN-LSTM), and CNN ensembled with bidirectional LSTM (CNN-BLSTM).

**Results:**

The aforementioned models are evaluated using metrics such as accuracy, precision, recall, and F1-score. The best performing algorithm, LSTM, has an overall accuracy of 98.82% and F1-score of 0.97.

**Discussion:**

The LSTM algorithm's extremely high predictive accuracy can be attributed to its penchant for capturing sequential patterns in time series based audio data. In summary, this algorithm is able to ingest patient audio recordings and make precise lung disease predictions in real-time.

## 1. Introduction

Anomaly detection in the field of computing for health and wellbeing has emerged as a prominent research topic, driven by the availability of vast amounts of medical data and the increasing need for accessible and scalable applications in real-world healthcare settings. The ability to leverage digital technologies, such as digital stethoscopes, has revolutionized the way respiratory audio files from patients' lungs are captured and analyzed. This paradigm shift opens up new possibilities for diagnosing lung ailments using advanced computational techniques. In this paper, we focus on the experimentation, detection, and classification of lung anomalies from respiratory audio files using deep-learning models with hyper-tuned neural networks. Our goal is to develop a robust and accurate model that can effectively diagnose patients based on their respiratory audio recordings.

The advent of digital stethoscopes has significantly transformed the medical landscape, enabling the collection of audio data that encompasses respiratory sounds. These digital audio files hold valuable information for diagnosing various respiratory conditions. For instance, the presence of wheezing sounds often indicates the occurrence of obstructive airway diseases, such as Chronic Obstructive Pulmonary Disease (COPD) (1). By harnessing the power of artificial intelligence algorithms, it becomes feasible to analyze these respiratory audio files and make diagnostic predictions using computational methodologies. Successful implementation of such applications could lead to the deployment of our software in hospitals nationwide, providing physicians with a diagnostic classifier that can support, validate, or further investigate their own clinical assessments.

In this paper, we tackle the formal problem of developing a robust deep-learning model consisting of hyper-tuned neural networks to diagnose lung ailments using respiratory audio files. This approach involves processing a large collection of audio files and providing accurate diagnoses of respiratory diseases such as bronchiolitis. To achieve this, we employ innovative deep-learning techniques to train our model, enabling it to effectively classify and predict respiratory anomalies.

To gain insights into the existing research landscape and inform our work, we conducted a thorough survey of relevant literature. Rocha et al. (2) contributed a comprehensive dataset comprised of 6898 respiration cycles extracted from 920 recordings obtained from 126 subjects. These respiratory cycles encompass various abnormalities, including crackles and wheezes. This dataset serves as a foundational reference for our own research. In a related context, Shin et al. (3) explored the utilization of cockpit audio data to detect significant events, presenting valuable strategies for handling noisy audio recordings and extracting meaningful features.

Furthermore, Acharya et al. (4) proposed a hybrid convolutional neural network (CNN) and recurrent neural network (RNN) model for crackle and wheeze classification, which aligns with our dataset and objectives. They achieved an accuracy of 66.31% with their algorithm. Kim et al. (5) demonstrated the effectiveness of CNN models in medical audio classification, providing valuable insights into the performance of CNNs on audio data. Similarly, Aykanat et al. (6) concluded that CNNs in combination with support vector machines (SVM) offer accurate classification and pre-diagnosis of respiratory audio. Their findings validate the potential of CNNs in our research domain.

In the pursuit of accurate classification, Fraiwan et al. (7) proposed a hybrid CNN-LSTM (long short-term memory) approach for medical audio data classification. Their model exhibited excellent performance, achieving high predictive accuracy. However, the details of the dataset used for classification and model development were not presented with sufficient clarity, posing a potential limitation to their work. Hsu et al. (8) utilized an open-source lung audio dataset they developed themselves, evaluating the classification results of eight different RNN variants. Their findings indicated that bidirectional models outperformed their unidirectional counterparts, providing valuable insights for our model selection and evaluation.

While the surveyed papers contribute significant insights to the field, it is essential to consider their limitations. Shin et al. (3) proposed algorithms that may not be highly scalable, potentially limiting their applicability in real-world scenarios with large-scale data. Acharya et al. (4) prioritized reducing memory costs over achieving higher model accuracy, which could impact the performance of their hybrid CNN-RNN model. Kim et al. (5) and Aykanat et al. (6) lacked rigorous parameter tuning for their deep learning algorithms, potentially limiting their overall performance. Fraiwan et al. (7), despite achieving high predictive accuracy, did not provide sufficient detail about the dataset used, which may hinder reproducibility and further investigation. Hsu et al. (8) acknowledged the need for additional research and experimentation to explore the performance of their state-of-the-art convolutional layers in depth. Finally, papers (9–16) contributed valuable insights into audio classification techniques, real-world applications, and data visualization methods, enriching our understanding of the broader context of audio classification in healthcare.

In conclusion, this paper aims to address the challenge of accurately diagnosing lung ailments by developing a robust deep-learning model that leverages respiratory audio files to perform disease detection and classification on patients. Through relevant literature surveys, our team has gained insights into various methodologies, datasets, and models proposed by previous researchers in this domain. By building upon their contributions, we aim to develop a highly accurate model that can effectively classify lung diseases and provide valuable diagnostic predictions.

## 2. Materials and methods

### 2.1. Data collection

Rocha et al. (2) developed the respiratory sound database that was used in this work with the intention of analyzing and contrasting various respiratory sound categorization algorithms. The recordings and annotations are the two main parts of the database, which is publicly available and accessible to everyone.

126 participants including healthy controls and individuals suffering from various lung conditions provided the recordings. Four clinical centers in Portugal, Greece, Turkey, and Serbia were used to find the participants. A digital stethoscope (Littmann 3200, 3M) connected via Bluetooth to a laptop computer was used to make the recordings. Following a predetermined methodology, the stethoscope was placed on the subjects' anterior, lateral, and posterior chest areas. The individual was required to sit still and breathe normally while the protocol called for recording respiratory sounds for 10 s in each place. Each participant underwent the protocol twice, yielding 920 recordings in all. The database for Respiratory Sound has a size of 2.01 GB total.

Two groups of specialists annotated the data: one for respiratory cycles and the other for events including crackles and wheezes. Three specialists from separate clinical centers annotated the breathing cycles, noting the beginning and conclusion of each inhalation and expiration cycle as well as the presence/absence of accidental sounds. Four experts from several clinical centers annotated the events, pinpointing where each crackle and wheeze occurred throughout each respiratory cycle.

The database was created for an international competition: IFMBE's International Conference on Biomedical and Health Informatics's first scientific challenge. The competition sought to advance work on automatic analysis of patient respiratory audio.

To assure accuracy and dependability of the source data, the data gathering method adhered to a number of ethical and technological standards. All participants' informed consent had to be obtained, their identity and confidentiality had to be maintained, and the Declaration of Helsinki's tenets had to be followed. Technical requirements included employing a consistent recording tool and process, providing a quiet atmosphere throughout the recordings, assessing the quality of the recordings before annotating, and safely storing the data.

There were a number of difficulties and restrictions in the data collection procedure for the Respiratory Sound Database. For example it was difficult to find enough individuals with various respiratory disorders from different clinical settings, which necessitated coordination and cooperation between researchers from many institutions and nations. Prior to annotation, training and calibration sessions were necessary to assure high inter-annotator agreement among specialists with various clinical backgrounds and expertise. The lack of recordings from other respiratory illnesses such as tuberculosis or lung cancer was a drawback of the data-gathering process. Another drawback was the absence of recordings from various body positions or breathing styles such as lying down, coughing, or deep breathing.

### 2.2. Feature engineering

For our research, features were extracted from each patient recording using speech and audio signal processing systems. Specifically, our team extracted the following five key features: mel-frequency cepstrum coefficients, chromagram, mel-scaled spectrogram, spectral contrast, and tonal centroids. We then stored the above results in numerical form via matrix arrays. These arrays capture critical information such as respiratory oscillations, pitch content, breathing amplitude, audio peaks/valleys, and chord sequences from the input audio files.

#### 2.2.1. Mel-frequency cepstrum coefficients

The mel-frequency cepstrum (MFC) constitutes the power spectrum of a sound. Taken together, MFCCs are coefficients that comprise the above sound spectrum. These coefficients are obtained by using linear cosine transform of a log power spectrum on a non-linear mel-frequency scale (17).

The mathematical formulation is shown below where *MFCCs*[*n*] represents the Mel-frequency cepstral coefficients for the *n*-th frame, IDCT refers to the Inverse Discrete Cosine Transform, *H*_*m*_[*k*] denotes the filterbank weights for the *m*-th Mel filter at frequency bin *k*, and *X*[*k*] represents the magnitude spectrum of the *k*-th frequency bin (18).


(1) $$\begin{array}{l} MFCCs\left[n\right]=\text{IDCT}\left(\log\left(\overset{M}{\underset{m=1}{\sum }}H_{m}\left[k\right]\cdot |X\left[k\right]{|}^{2}\right)\right) \end{array}$$

The MFCC values for each patient audio file is derived by first calculating the fourier transform of the individual's respiratory audio. The resulting power spectrum output is then mapped onto the mel scale using cosine overlapping windows. At each mel frequency point, the log of powers is calculated followed by discrete cosine transforms on each log power value. This feature extraction procedure ultimately produces MFCC amplitude values.

#### 2.2.2. Chromagram

Chromagrams map audio pitches into a single octave, comprised of 12 semitones. Our team extracted chroma features from each patient's respiratory audio recording by deploying a combination of Q Transform and Short-Time Fourier Transform (STFT) on each ingested file. These specialized features capture the tonal spectrum of patients' respective audio waveforms by mapping each pitch to one of twelve possible semitones. This enables subsequent high-level analysis such as chord recognition, structural audio analysis, and harmonic similarity measurements.

A chromagram can be formulaically expressed via the equation below (18).


(2) $$\begin{array}{l} \text{Chromagram}\left(t,c\right)=\underset{\text{all frames}i}{\sum }|\text{STFT}\left(t,f_{i}\right)|\cdot \delta \left(\text{Pitch}\left(f_{i}\right)-c\right) \end{array}$$

In the above formula *t* represents the time frame index, *c* represents the chroma (pitch class) index, *STFT*(*t, f*) represents the Short-Time Fourier Transform magnitude at time frame *t* and frequency bin *f*_*i*_, pitch represents the estimated pitch corresponding to frequency bin *f*_*i*_, δ is the Dirac delta function, which returns 1 if the condition inside the parentheses is true and 0 otherwise (18).

#### 2.2.3. Mel-scaled spectrogram

The Mel-scaled spectrogram visually displays a time series audio file as a 2-dimensional image. In this context, time is on the x-axis while frequency is on the y-axis. A particular point in time inside the sound file corresponds to a single pixel's brightness inside its corresponding image.

Conceptually speaking, Fast Fourier Transforms (FFTs) are applied to each condensed frame of a patient's respiratory audio. This process results in a frequency band spectrum output. The spectrum is pushed through a frequency-domain filter bank responsible for transforming our sound data onto the mel-scale. Higher mel-scale values correspond to greater pixel intensity inside the image.


(3) $$\begin{array}{l} S_{\text{mel}}\left(t,f\right)=\overset{M}{\underset{m=1}{\sum }}H_{m}\left(f\right)\cdot |S\left(t,f\right)| \end{array}$$

In the above formula, *S*_mel_(*t, f*) represents the Mel Spectrogram at time *t* and frequency *f*. *S*(*t, f*) captures the magnitude spectrum of the audio signal at time *t* and frequency *f*. *H*_*m*_(*f*) denotes the filter bank response at frequency *f* for the *m*_*th*_ mel filter, and *M* represents the total number of mel filters used (18).

#### 2.2.4. Spectral contrast

Spectral contrast is defined as the decibel difference between peaks and valleys in an audio spectrum (19). The objective of this feature extraction technique is to analyze the contrast in frequency bands over a harmonic spectrum to quantify perceived decibel differences. Our team calculated spectral contrast in patient respiratory audio files using logarithmic spectral differences.

The corresponding equation is shown below (18).


(4) $$\begin{array}{l} \text{Spectral Contrast}\left(X\right)=\frac{1}{N}\sum_{i=1}^{N}|\log_{10}\left(X_{i}\right)-\frac{1}{M}\sum_{j=i-L}^{i+L}\log_{10}\left(X_{j}\right)| \end{array}$$

In this formula, *X* represents the magnitude spectrum of the audio signal while *X*_*i*_ is the magnitude at frequency bin *i* within a specific frequency band (18). *N* is the total number of frequency bins considered and *M* is the number of neighboring frequency bins used to calculate the average magnitude (18). Finally, *L* represents the half-size of the range of neighboring frequency bins.

#### 2.2.5. Tonal centroids

Tonal centroids can be interpreted as the resting centers of a pitch or chord. Taken together these centroids help quantify the central pitches of an audio sequence. They are able to effectively summarize both the characteristics and tonal movements of respiratory audio files over time.

The mathematical formulation is shown below where *p*_*i*_ represents the pitch class (0 to 11) and *f*_*i*_ represents the frequency of that pitch class within the audio (18). The sum is taken over all 12 pitch classes, and the resulting value represents the tonal centroid (18).


(5) $$\begin{array}{l} \text{Tonal Centroid}=\frac{\overset{11}{\underset{i=0}{\sum }}\left(f_{i}\times p_{i}\right)}{\overset{11}{\underset{i=0}{\sum }}f_{i}} \end{array}$$

While tonal centers are most frequently deployed in musical analysis, they have proven useful within the context of dissecting patient breathing audio as well. In particular, our team has been able to extract tonal center values associated with patient coughing, wheezing, and lung crackling noises from recorded audio.

### 2.3. Process flow

**Figure 1:**
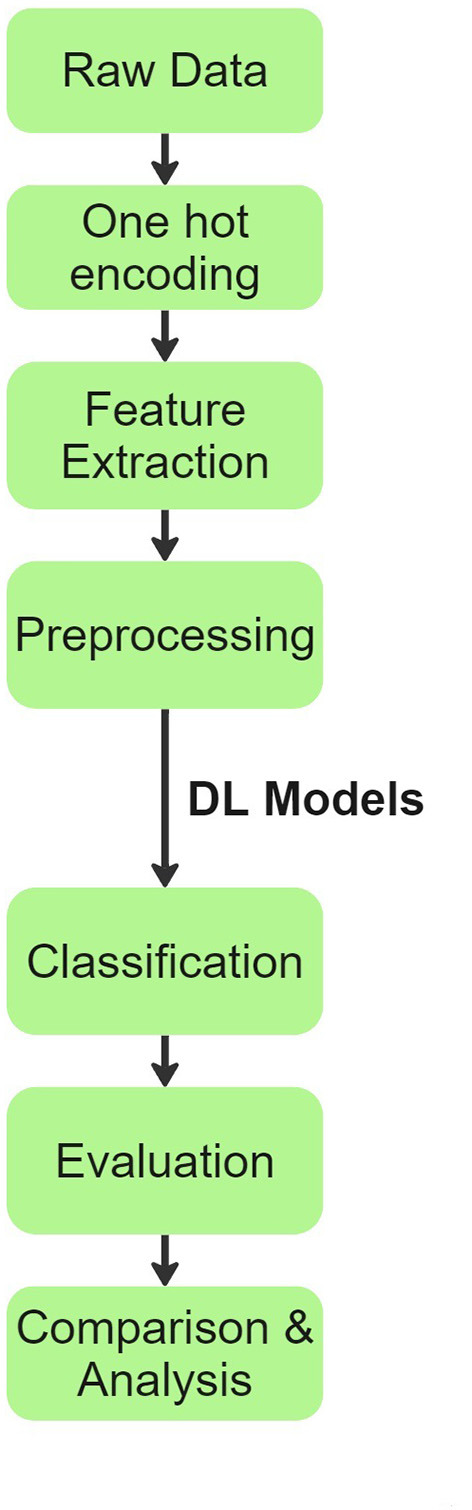
Process flow chart explaining the methodology inspired from (20).

Our team's overall process flow is visually summarized in Figure 1. Initially, raw patient audio recordings and corresponding annotations were attached together for preprocessing. In total, there are 920 distinct audio files obtained from 126 patients. Each patient has only one disease classification label. The original distribution of diseases across patients and their audio files is shown below:

- Patients {Asthma: 1, Bronchiectasis: 16, Bronchiolitis: 13, COPD: 785, Healthy: 26, LRTI: 2, Pneumonia: 6, URTI: 14}
- Audio Recordings {Asthma: 1, Bronchiectasis: 16, Bronchiolitis: 13, COPD: 795, Healthy: 35, LRTI: 2, Pneumonia: 35, URTI: 23}

Disease labels were given numerical values from 0 to 7 with “Chronic Obstructive Pulmonary Disease (COPD)” referring to 0, “Healthy” referring to 1, “Upper Respiratory Tract Infections (URTI)” referring to 2, “Bronchiectasis” referring to 3, “Pneumonia” referring to 4, “Bronchiolitis” referring to 5, “Asthma” referring to 6, and “Lower respiratory tract infection (LRTI)” referring to 7.

Prior to cleaning raw data, the one-hot encoding procedure is applied to transform relevant categorical variables. Each category is turned into a binary vector in this encoding technique, with the exception of the element corresponding to the category itself which is set to one. During preprocessing, Asthma and LRTI were removed due to very low counts in the source dataset. In the data exploration stage, our team also noticed over 80% of actual patient diagnoses fell within the COPD class. We used the imbalanced-learn (21) toolbox to oversample minority diseases and undersample the majority representation (COPD) to create a more balanced dataset for subsequent model training and patient disease classification.

Applying a combination of over and under-sampling techniques from the aforementioned library, our team was able to generate an updated input dataset with less imbalanced sample sizes across all six diseases. See distribution below:

- Audio Recordings {Bronchiectasis: 73, Bronchiolitis: 63, COPD: 393, Healthy: 118, Pneumonia: 118, URTI: 82}

After completing data pre-processing activities, 5 features (mel-frequency cepstrum coefficients, chromagram, mel-scaled spectrogram, spectral contrast, and tonal centroids) were extracted from each individual patient recording using a python library called librosa (22). These features captured critical information such as respiratory oscillations, pitch content, amplitude of breathing noises, peaks and valleys in audio, and chord sequences from the sound recordings. Feature extraction is described in detail in Section 2.2 of this paper. The results are then stored in 2 patient delineated arrays, one consisting of extracted features from raw audio files and the other containing corresponding disease labels.

**Figure 2:**
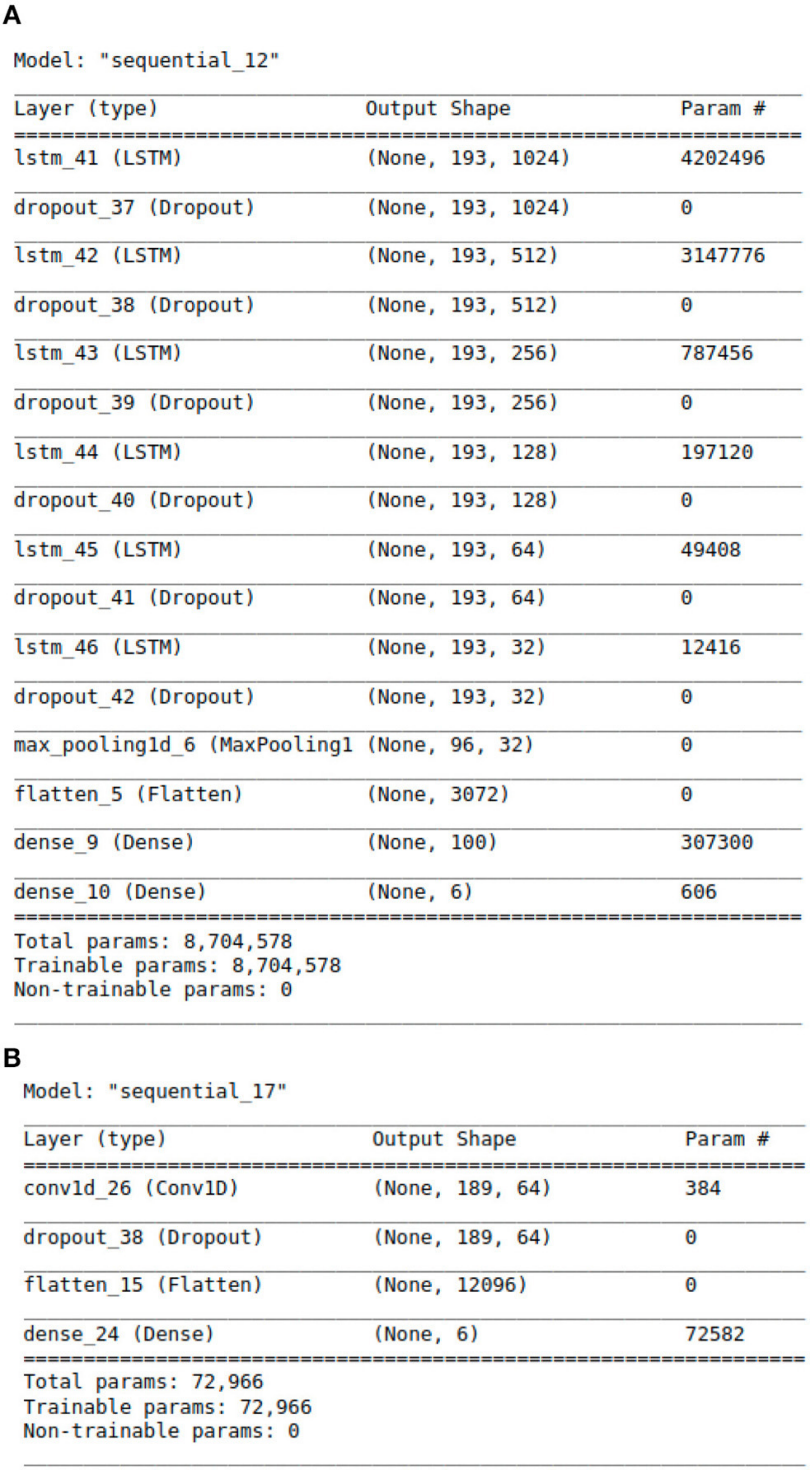
The Architecture showing the specific layers and the parameters of the models. **(A)** This is the LSTM model. **(B)** This is the CNN model.

**Figure 3:**
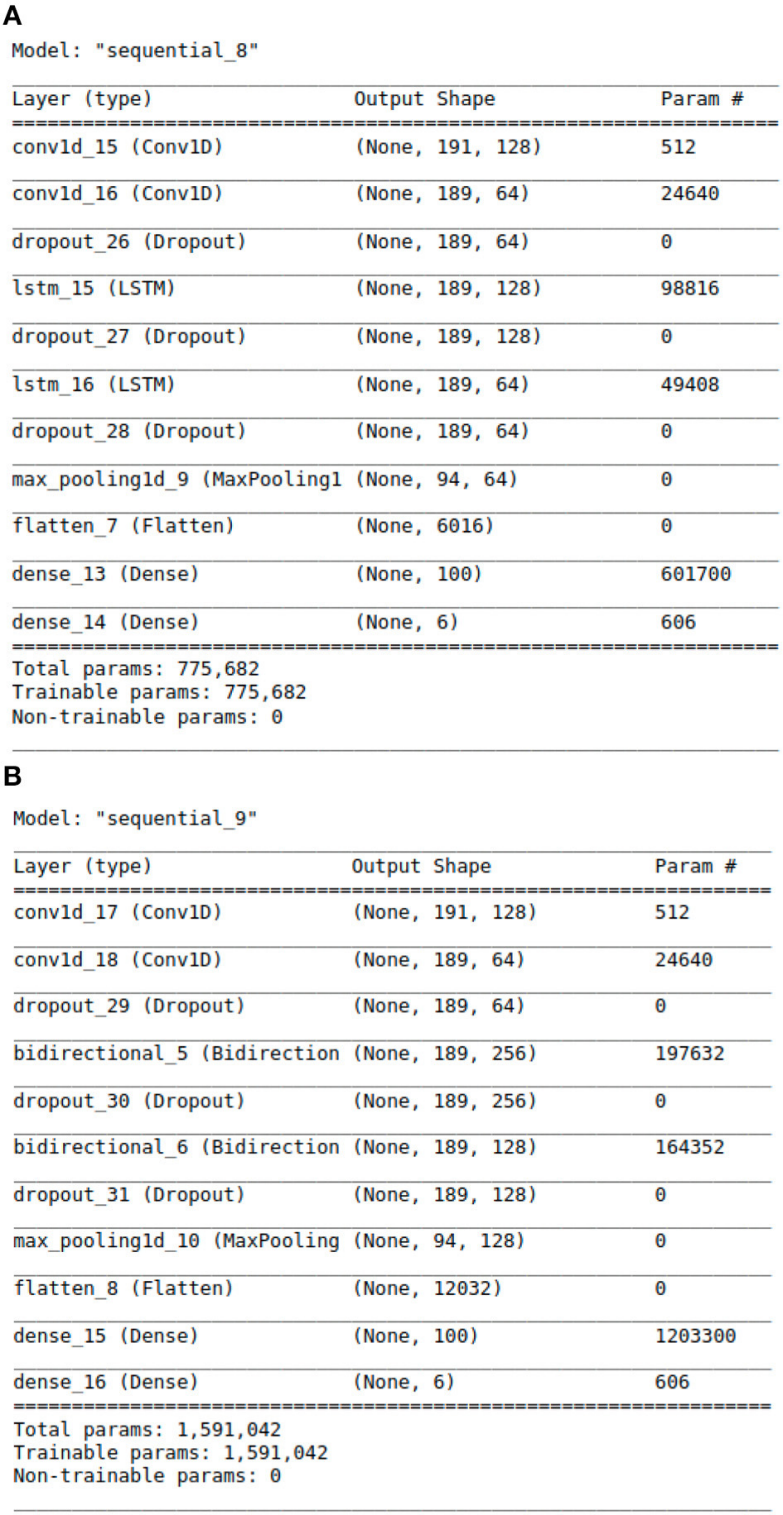
The Architecture showing the specific layers and the parameters of the models. **(A)** This is the CNN-LSTM model. **(B)** This is the CNN-BLSTM model.

With above steps completed, the aforementioned data arrays were segmented into train and test datasets following an 80:20 split. This was done using Python's Scikit-learn (23) library. The data was then passed to the deep learning models for training and validation. For modeling purposes, CNN, LSTM, CNN ensembled with unidirectional LSTM, and CNN ensembled with bidirectional LSTM models were implemented. Our team experimented with the 4 neural networks' layering structures, tuned hyper-parameters, selected model checkpoint values, and calculated early stopping parameters for best classification results. Additionally, we tested a range of plausible values for every model parameter across all four neural networks. The algorithms were designed using Python libraries Tensorflow (24) and Keras (25). The libraries Numpy (26) and Pandas (27) were also used for vectorization and data manipulation, respectively. The exact architectural structure of our deep learning models can be found in Figures 2A, B, 3A, B.

### 2.4. Models

#### 2.4.1. Convolutional neural network

Convolutional Neural Networks (CNNs) are a class of deep learning models widely used in image and audio analysis. They are particularly effective in extracting spatial patterns and features from data. In the context of respiratory audio recordings, CNNs can learn to identify distinctive patterns, such as wheezes and crackles, which are essential for diagnosing lung diseases. CNNs use convolutional layers to convolve filters over the input data, followed by activation functions and pooling layers to reduce spatial dimensions. This process enables the network to learn hierarchical representations of the input data, making CNNs a popular choice for audio classification tasks (28).

#### 2.4.2. Long short-term memory

Long Short-Term Memory (LSTM) is a type of recurrent neural network (RNN) specifically designed to capture long-term dependencies in sequential data. Unlike traditional RNNs, LSTM has a gating mechanism that allows it to retain important information for an extended period while discarding irrelevant information (29). In the context of respiratory audio recordings, LSTM can effectively model sequential patterns, such as respiratory oscillations and irregularities over time, which are critical for diagnosing respiratory illnesses. LSTM's ability to learn from long-range dependencies makes it suitable for time-series data like audio signals.

#### 2.4.3. Convolutional neural network with long short-term memory

CNN-LSTM is a hybrid model that combines the strengths of CNNs and LSTMs. In this architecture, the initial layers of the model use CNNs to extract spatial features from the input data. The output of the CNN layers is then fed into LSTM layers to capture temporal dependencies and sequential patterns present in the data (30). This combination allows the model to effectively process both spatial and temporal information, making it well-suited for tasks involving sequential data, such as respiratory audio recordings.

#### 2.4.4. Convolutional neural network with bidirectional long short-term memory

CNN-BLSTM is another hybrid model that combines CNNs with Bidirectional LSTMs. Similar to CNN-LSTM, CNN-BLSTM uses CNN layers for spatial feature extraction. However, in the subsequent layers, bidirectional LSTMs are employed to process the data bidirectionally, allowing the model to access both past and future information in the sequential data. This enables the model to gain a deeper understanding of the temporal dynamics and dependencies in the respiratory audio recordings, resulting in improved accuracy for diagnosing lung diseases (31).

Lung audio data is sequential and exhibits patterns over time. LSTM models are a type of recurrent neural network (RNN) that can process sequential data, such as audio signals, by maintaining a hidden state that encodes the temporal dependencies in the input sequence (32). LSTM models have a special structure that allows them to avoid the vanishing or exploding gradient problem that plagues conventional RNNs. It has memory cells that can store information over long periods and gates that control the flow of information into and out of the memory cells (33). Lung diseases may manifest as subtle, long-term changes in audio patterns. LSTMs excel at capturing long-term dependencies in data, making them capable of identifying these complex, nuanced patterns (7).

CNN models are a type of feedforward neural network that can extract spatial features from the input data by applying convolutional filters and pooling operations (34). CNNs, by default, capture short-range dependencies due to their local receptive fields, and they may struggle with capturing longer-term trends. CNN models are primarily designed for and are good at handling high-dimensional and structured data, such as images, but they do not have the ability to model temporal dependencies in sequential data, such as audio signals (8).

CNN-LSTM and CNN-BLSTM models are hybrid models that combine CNN and LSTM layers to leverage the advantages of both techniques. CNN-LSTM models use a unidirectional LSTM layer after the CNN layer to process the extracted features sequentially. CNN-BLSTM models use a bidirectional LSTM layer after the CNN layer to process the extracted features from both directions (forward and backward).

The best performing algorithm classifies each patient's respiratory audio with one of the following diagnoses: COPD, Healthy, URTI, Bronchiectasis, Pneumonia, or Bronchiolitis. The model's classification results are evaluated with precision, recall, F1-score, and accuracy metrics. Deploying these deep learning models on respiratory audio data allows for more accurate and efficient diagnosis of lung diseases, ultimately benefiting patients and healthcare practitioners alike.

### 2.5. Performance metrics

A number of performance criteria such as accuracy, precision, F1-score, and recall were used to assess model performance. Each of these metrics offers insightful information about a model's predictive prowess.

#### 2.5.1. Accuracy

The accuracy metric provides a sense of how well a classification algorithm performs overall. It shows the percentage of instances that were accurately categorized relative to all instances. The following equation can be used to calculate accuracy (35):


(6) $$\begin{array}{l} \text{Accuracy}\\ =\frac{\text{True Positives}+\text{True Negatives}}{\text{True Positives}+\text{True Negatives}+\text{False Positives}+\text{False Negatives}} \end{array}$$

#### 2.5.2. Precision

A measure of a model's accuracy in identifying positive cases is called precision. In other words, it is the proportion of genuine positives to the total of both true and false positives (35). The following equation can be used to determine precision (35):


(7) $$\begin{array}{l} \text{Precision}=\frac{\text{True Positives}}{\text{True Positives}+\text{False Positives}} \end{array}$$

#### 2.5.3. Recall

The capacity of the model to accurately detect positive cases is measured by recall, often referred to as sensitivity or true positive rate. It measures the proportion of real positives to the total of real positives and real negatives (35). The following equation can be used to determine recall (35):


(8) $$\begin{array}{l} \text{Recall}=\frac{\text{True Positives}}{\text{True Positives}+\text{False Negatives}} \end{array}$$

#### 2.5.4. F1-score

A balanced indicator of a model's performance, the F1-score is a harmonic mean of precision and recall (35). It combines the precision and recall values into a single score after taking both into account (35). The following equation can be used to determine the F1-score (35):


(9) $$\begin{array}{l} \text{F1-score}=2\times \frac{\text{Precision}\times \text{Recall}}{\text{Precision}+\text{Recall}} \end{array}$$

The process of selecting the optimal model and architecture involved a systematic and empirical approach. It encompassed a rigorous evaluation using the specific dataset, wherein a diverse range of layer structures and parameter configurations were explored. The primary objective was to ascertain the architecture that exhibits superior performance concerning critical metrics such as accuracy, precision, recall, and F1-Score in the context of lung disease detection from respiratory audio data.

### 2.6. Novelty

Our research's focus on applying LSTM algorithms to patient respiratory audio files offers a novel approach to pulmonary disease diagnostics.

#### 2.6.1. Long-term dependencies

The LSTM architecture is specifically designed to address the vanishing gradient problem in traditional RNNs, which hinders the modeling of long-term dependencies in sequential data (29). In the context of respiratory audio data, where crucial diagnostic information may span over multiple time steps, LSTM's ability to capture long-term dependencies becomes paramount (29). This allows the model to better discern complex patterns and variations in respiratory sounds, leading to more accurate disease classification.

#### 2.6.2. Sequential context understanding

In respiratory audio data, the context of each audio segment is crucial for accurate diagnosis. LSTM excels in learning sequential context by maintaining an internal memory cell and carefully regulating information flow through gate mechanisms. This mechanism allows the LSTM model to store relevant information from past audio segments and selectively integrate it into the current processing, enabling a more comprehensive understanding of the audio data (36).

The proposed LSTM model shown in Figure 2A has 8,704,578 parameters, 6 LSTM layers and a total of 16 layers, whereas the hybrid models only have 2 LSTM layers. The additional layered complexity results in greater accuracy. The proposed LSTM model that achieves the highest performance has the most complex architecture with the largest number of parameters.

The paper on the Universal Law of Robustness via Isoperimetry by Bubeck theoretically affirms that a model with an increased number of layers possesses greater capacity to effectively learn and retain complex patterns, consequently enabling the potential to encompass a larger repertoire of mapping functions by virtue of having a larger number of layers and parameters (37). The complexity of the LSTM architecture allows it to model complex temporal dynamics in audio signals, which is essential for accurate audio signal processing (38).

Another key novel step in the learning process of LSTM networks is backpropagation, a technique for calculating the gradient of a loss function with regard to the network weights for a single input-output example. Local gradients are computed at each stage of the backpropagation process, accumulated, and then back propagated to earlier time steps. Backpropagation Through Time (BPTT) is a term that is frequently used to describe this phenomenon. However, BPTT using conventional RNNs might result in gradients that vanish or explode. With their distinctive architecture, LSTMs solve this issue by allowing gradients to continue to flow across numerous time steps without disappearing or blowing up, allowing the network to learn from longer sequences (39).

## 3. Results

Table 1 presents a comparative summary of predictive performance among our four deep learning algorithms: LSTM, CNN, CNN-LSTM, and CNN-BLSTM. These models are assessed using evaluation criteria such as Accuracy, Precision, Recall, and F1-Score. The model training time is also included in our table to show relative execution time.

**Table 1:** Accuracy, training time/epoch, precision, recall, and F1-score of different models.

**Model name**	**Accuracy (%)**	**Training time/epoch (s)**	**Precision**	**Recall**	**F1-Score**
LSTM	98.82	3.005	0.96	0.99	0.97
CNN	87.64	0.000078	0.83	0.82	0.81
CNN-LSTM	97.05	5.008	0.93	0.95	0.94
CNN-BLSTM	97.64	11.016	0.95	0.96	0.96

The LSTM model produced the highest scores across all four evaluation metrics. As such, it is our best performing algorithm. Its overall predictive accuracy sits at 98.82%. In comparison, the other 3 algorithms achieved accuracy levels of 97.64% for CNN-BLSTM, 97.05% for CNN-LSTM, and 87.64% for CNN. Given our adjusted input dataset's imbalanced class distribution, the F-1 Score serves as a more robust metric to evaluate algorithmic performance due to its consideration of both Precision and Recall. From this standpoint LSTM also outperforms its competitors. As shown in Table 1, the F-1 Scores for LSTM, CNN-BLSTM, CNN-LSTM, and CNN are 0.97, 0.96, 0.94, and 0.81 respectively.

According to the literature, LSTM models perform better than CNN, CNN-LSTM, and CNN-BLSTM models for lung disease detection from lung audio signals because: LSTM models can capture the temporal dynamics and variability of lung sounds better than CNN models, which only focus on the spatial features. LSTM models are more robust and can handle noisy and corrupted lung sounds better than CNN models, which are sensitive to noise and distortion. LSTM models can generalize better to unseen data and different lung diseases than CNN models, which tend to overfit and have poor transferability. LSTM models can outperform CNN-LSTM and CNN-BLSTM models, as the advantages of CNNs in spatial data processing are not exploitable with audio signal processing.

**Figure 4:**
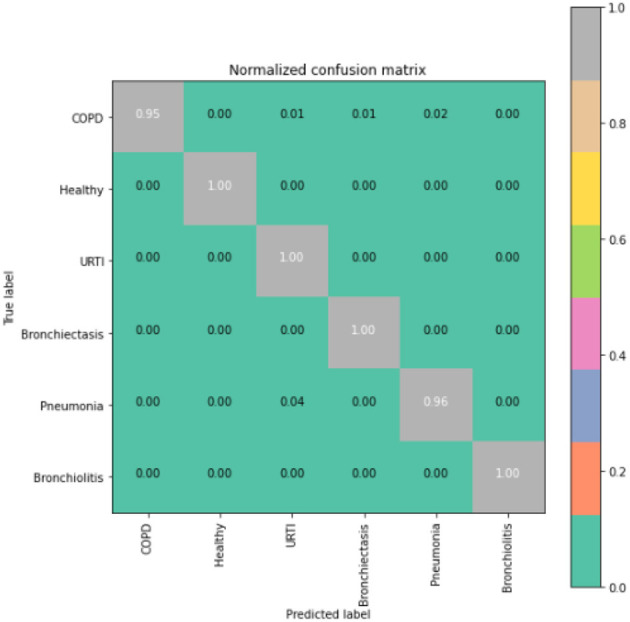
LSTM confusion matrix.

**Figure 5:**
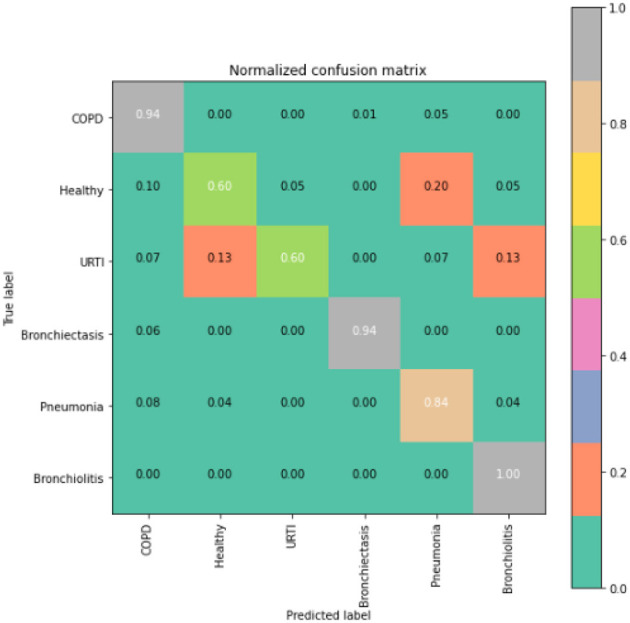
CNN model confusion matrix.

**Figure 6:**
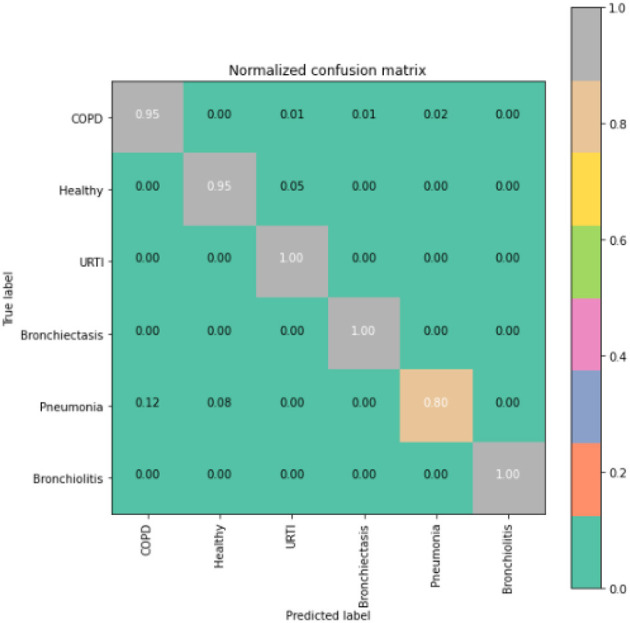
CNN-LSTM confusion matrix.

**Figure 7:**
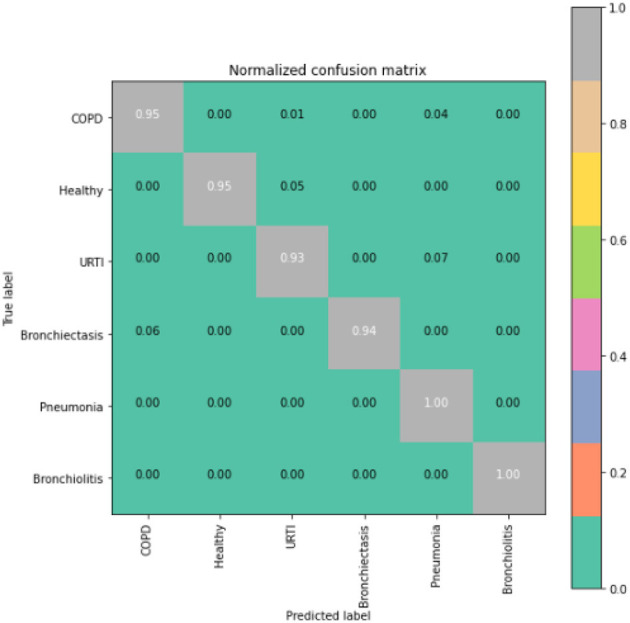
CNN-BLSTM model confusion matrix.

To gain deeper insights into the results, we also supply the output confusion matrix for each respective model (LSTM in Figure 4, CNN in Figure 5, CNN-LSTM in Figure 6, and CNN-BLSTM in Figure 7). Confusion matrices compare an algorithm's predicted labels against the true labels for every lung disease category comprising the response variable. By examining the true positive (TP), false positive (FP), true negative (TN), and false negative (FN) outcomes, we see that the LSTM model performs exceptionally well across all lung disease classifications. For example, it was able to accurately predict all cases of healthy, URTI, bronchiolitis, and bronchiectasis patients. Additionally, the remaining two diseases (pneumonia and COPD) were accurately classified 96% and 95% of the time, respectively.

The runner up algorithm, CNN-BLSTM, also performed well across all lung disease classifications. As evidenced in Figure 7, it was able to predict all categories of lung diseases and healthy controls at a rate of 93% or higher. But it underperformed LSTM by 5–7 percentage points for healthy, URTI, and bronchiectasis patients. Finally, CNN was our worst performing algorithm overall. It struggled to distinguish between healthy patients and those suffering from upper respiratory tract infections and pneumonia.

## 4. Discussion

The domain of machine learning classification in healthcare is currently ripe for exploration. An overwhelming amount of data is being collected and stored in many different medical fields. Within the field of pulmonology, some physicians are utilizing digital stethoscopes to record patient respiratory cycles for diagnostic purposes. These audio recordings are used to help detect and confirm irregular breathing patterns such as wheezes and crackles which may be indicative of certain lung diseases. The purpose of our research is to develop a robust machine learning tool to aid physicians in their pulmonary diagnostic endeavors. Timely and correct diagnosis is crucial for effective treatment, making deep learning methods cost-effective and time-efficient for both patients and practitioners.

Several other studies have also used deep learning algorithms to classify patient respiratory audio files (4, 5, 8). For example, Kim et al. (5) attained an accuracy score of 86.5% using convolutional neural networks to categorize 1918 respiratory sounds recorded in the clinical setting. Acharya et al. (4) achieved an accuracy of 71.81% using a CNN-RNN hybrid model to identify breathing sound anomalies for automated diagnosis of respiratory diseases. In contrast, our team's LSTM model reaches an improved accuracy of 98.82%, indicating its potential for clinical use.

In comparison to the LSTM model, the CNN-BLSTM algorithm presents a possible alternative approach. By integrating spatial information extraction from CNN convolutional layers and temporal dependency modeling through bidirectional LSTM layers, it combines the strengths of both CNN and LSTM. This unique architecture allows the model to access both past and future information, enhancing its understanding of the input data.

Despite its upside, this study does have certain limitations that should be addressed in future iterations. For example, the input dataset was heavily skewed toward COPD, the most prominent class. To address this imbalance, we employed oversampling and undersampling techniques to balance the training set. While oversampling can be helpful, it introduces some bias into the model. Moving forward, we would like to curate a more balanced dataset that encompasses high-quality audio data from a diverse body of patients. Future studies would benefit from an extensive data gathering stage to ensure a comprehensive and representative dataset.

A major contributor to our LSTM model's high predictive accuracy is rigorous feature engineering. Prior studies like Kim et al. (5) and Acharya et al. (4) leveraged mel-spectrogram to convert input audio files into images for classification. Moreover, Hsu et al. (8) used spectrogram, mel-frequency cepstrum coefficients, and energy summation to enable adventitious sound detection. Our model built upon prior research to deploy a combination of MFCC, chromagram, mel-scaled spectrogram, spectral contrast, and tonal centroid input features for algorithm training. This specific combination of feature variables captured critical information such as respiratory oscillations, pitch content, breathing amplitude, audio peaks/valleys, and chord sequences from input audio files. Although integrating the aforementioned features increases model complexity compared to peer papers, it succeeds in boosting overall predictive accuracy.

In addition to feature engineering respiratory audio, our team also calibrated and tuned over 8 million model parameters, leading to our finalized LSTM algorithm (see Figure 2A). The algorithm consists of approximately 16 layers total. Each layer takes a 3D tensor as input with the following dimensions: batch_size, time steps, input_features. The output shape of each LSTM layer is (none, 193, n). 193 represents the number of time steps in the input sequence while “n” denotes the number of LSTM units in each layer. Six dropout layers are deployed to prevent model over fitting by randomly dropping nodes from the previous LSTM layer. After the last LSTM layer, a 1D max-pooling layer reduces time steps by selecting the maximum value from a set. The output shape then becomes (none, 96, 32) with 96 time steps and 32 features. Following max pooling, a flatten layer converts the 3D tensor into a 1D tensor with 3,072 elements. Two dense layers follow the flatten layer for classification purposes. The first dense layer has 100 neurons while the second has 6 neurons, representing the 6 possible lung disease classifications our algorithm is capable of predicting.

An area of our work that warrants further exploration is neural network quantization. Quantization is a process that takes the weights, biases, and activation functions established during training and converts the corresponding 32-bit floats to 8-bit integers. This can significantly reduce a model's memory footprint while still maintaining state-of-the-art accuracy. Using a quantization model, we can theoretically deploy our real-time diagnostic tool in resource constrained platforms such as cell phones or tablets.

## Data availability statement

The Respiratory Sound Database used for experimentation in this paper can be found at the International Conference on Biomedical Health Informatics (ICBHI) 2017 (1).

## Author contributions

PZ: Conceptualization, Writing—original draft, Writing—review & editing, Formal analysis, Funding acquisition, Investigation, Methodology, Project administration, Software, Supervision, Validation, Visualization. AS: Conceptualization, Formal analysis, Investigation, Methodology, Software, Validation, Visualization, Writing—original draft, Writing—review & editing, AU: Conceptualization, Formal analysis, Investigation, Software, Validation, Writing—original draft, Writing—review & editing.
