# Supplementary material for: Pulmonary disease detection and classification in patient respiratory audio files using long short-term memory neural networks
Source: Front Med (Lausanne). 2023 Nov 3;10:1269784. doi: 10.3389/fmed.2023.1269784 (PMC10656606; doi:10.3389/fmed.2023.1269784)
Supplement: Supplementary file 1 [file Data_Sheet_1.PDF]

Supplementary Material

1 SUPPLEMENTARY DATA

No supplementary data available.

2 SUPPLEMENTARY TABLES AND FIGURES

2.1 ChatGPT Prompts to Generate Latex Math Formulas

Section 2.2 of the paper describes five feature engineering techniques used to extract key features from audio files. Within this section, mathematical formulas are provided for each technique. The LaTeX syntax behind these equations were generated by ChatGPT, but verified by our team for accuracy.

2.2 Figures

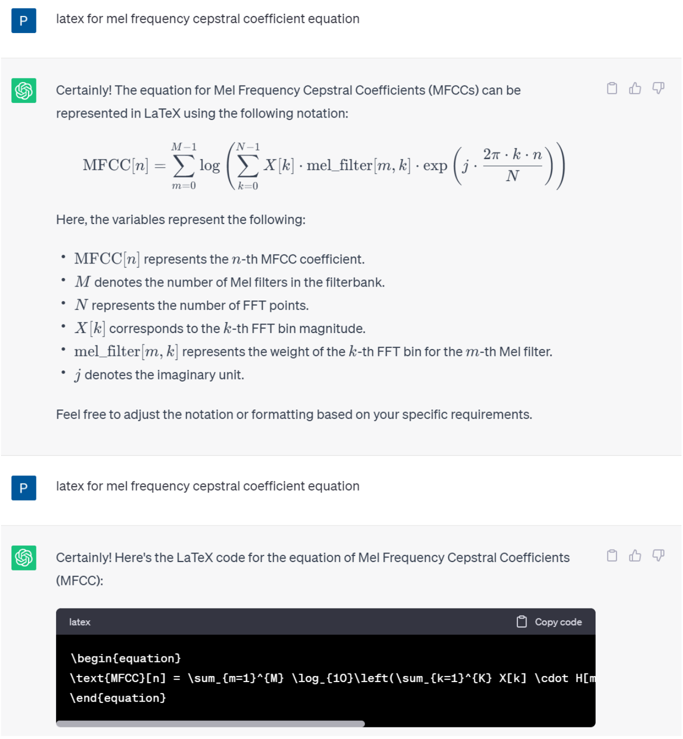

Figure S1. ChatGPT prompt and response for Mel-frequency Cepstrum Coefficients (MFCCs) LaTex equation syntax.

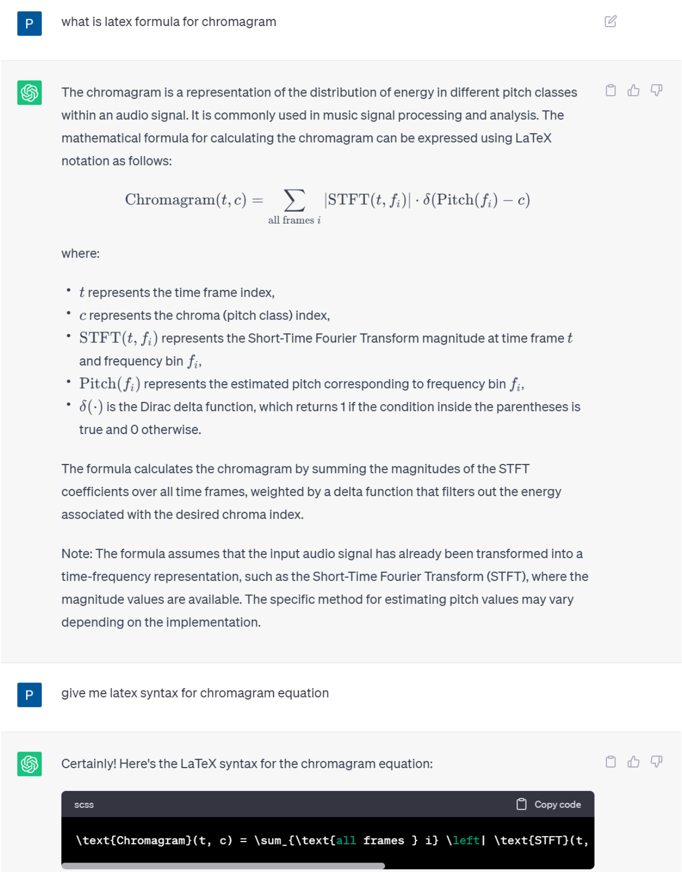

Figure S2. ChatGPT prompt and response for Chromagram LaTex equation syntax.

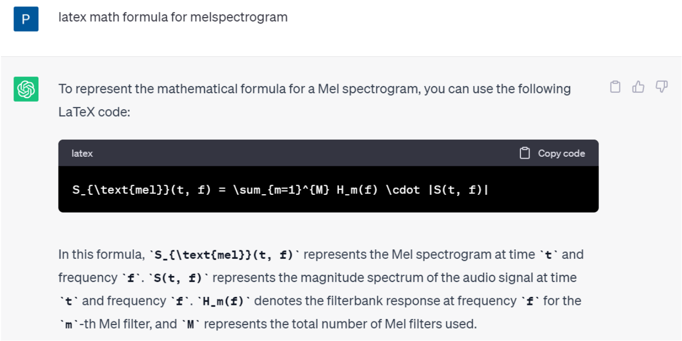

Figure S3. ChatGPT prompt and response for Mel-Scaled Spectrogram LaTex equation syntax.

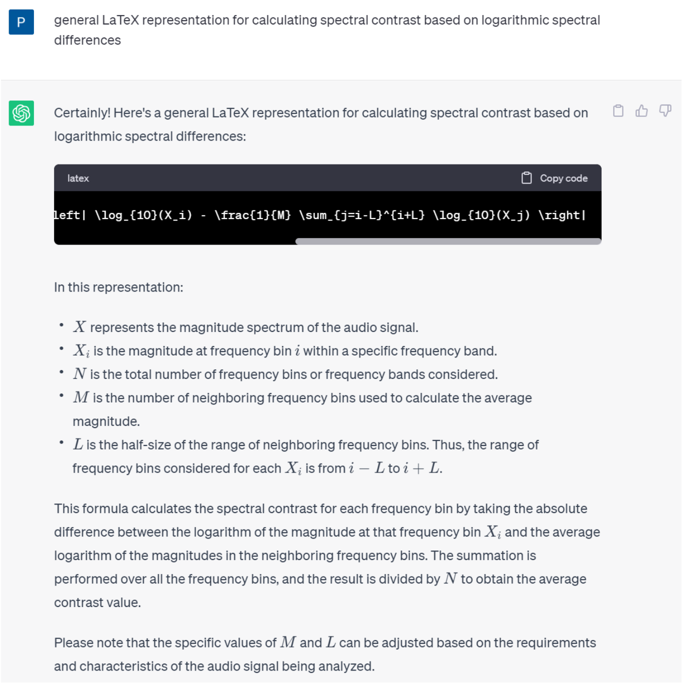

Figure S4. ChatGPT prompt and response for Spectral Contrast LaTex equation syntax.

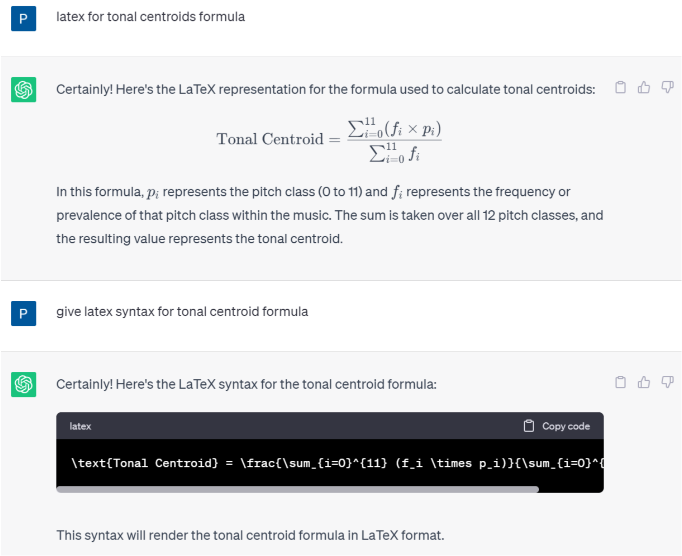

Figure S5. ChatGPT prompt and response for Tonal Centroids LaTex equation syntax.
